# Supplementary material for: Learning curve and surgical outcome of robotic assisted colorectal surgery with ERAS program
Source: Sci Rep. 2022 Nov 29;12:20566. doi: 10.1038/s41598-022-24665-w (PMC9709162; doi:10.1038/s41598-022-24665-w)
Supplement: Supplementary file 2 — Supplementary Information 2. [file 41598_2022_24665_MOESM2_ESM.zip › RAL Raw data-Ñ[▒K/051/Patho_ñw╝╨░O▒Kñσ.pdf]

臺中榮民總醫院 臺中市臺灣大道四段1650號 病理部活體組織切片 臨床醫學實驗及病理診斷中心 檢驗(查)報告

[redacted]

Pathologic diagnosis:
1. Rectum, robot low anterior resection --- Chronic ulcer, mild inflammation and focal fibrosis of the lamina propria. The bilateral cut ends and perirectal lymph nodes (0/35) are free of tumor. See Comment.
2. IMA root, dissection --- Unremarkable fibrovascular tissue and lymph node (0/1).
3. Rectum, cut end, distal, resection --- Chronic inflammation with focal fibrosis of lamina propria.

Ancillary study for diagnosis:
1. Deep cut done on section E.
2. Special stain for masson trichrome done on section D.
3. IHC stains for AE1/AE3 (highlight epithelial cells) and desmin (highlight smooth muscle) done on section D.

Gross description:
The specimen consists of 1) a segment of colon, 13 cm in length and 3.5 cm in proximal cut end circumference, 6 cm in distal circumference, and perirectal soft tissue. An ulcerative lesion, 1.8x1.1 cm, is noted, 2 cm to the nearest cut end. On cross section, no definite invasion is seen grossly. The both cut ends are free of tumor grossly. 2) IMA root, a piece of tan yellow soft tissue, 2.8x2.6x1.5 cm. 3) distal cut end, a piece of tan mucosa-covered soft tissue, 2x1.1x0.8 cm. Representative part for section: A) proximal cut end B) distal cut end C-F) lesion G-H) specimen 2-3 J-M) lymph nodes.

Comment:
The histologic picture of the non-ulcerative mucosa is reminiscent of localized ischemic change of the mucosa. The possibility of previous trauma, drug effect, infection or mucosal prolapse may be considered. Further clinical correlation is suggested.

Note:
The diagnosis is concurred in intradepartment consensus meeting on 2021/01/22.
#T-59600_2 #M-38000_2 0 1105 00CM00
#T-C4560_2 #M-09183_2 0 1105 000000

已電子簽章

敬告：本單僅供醫師參考使用，不具任何申請效力！！
請病患妥為保管，若不慎遺失需自負個人資料外洩責任！
